# Invariance of the Household Food Insecurity Access Scale Across Different Groups of Adolescents and Young Adults

**DOI:** 10.1177/03795721211019634

**Published:** 2021-06-15

**Authors:** Rainier Masa, Anjalee Sharma

**Affiliations:** 1 2331 The University of North Carolina at Chapel Hill, NC, USA; 2 Global Social Development Innovations, Chapel Hill, NC, USA

**Keywords:** household food insecurity, Ghana, adolescents, south Africa, food security

## Abstract

**Background:**

Cross-group comparisons of household food insecurity and its associations using multiple-item scales assume that scale scores can be interpreted as identical across groups. However, scores should not be interpreted as identical across groups without evidence of measurement invariance. Noninvariant measures indicate that the underlying construct may be different across groups.

**Objective:**

To determine whether the Household Food Insecurity Access Scale (HFIAS) is invariant across different groups of Ghanaian and South African youth aged 15 to 24.

**Methods:**

We analyzed cross-sectional quantitative data from 1437 and 4165 young South Africans and Ghanaians, respectively. Multi-group confirmatory factor analysis was used to examine whether the HFIAS was invariant across different groups of youth, including sex (male or female), age group (middle adolescence, late adolescence, or emerging adulthood), and receipt of child support grant (yes or no). We assessed 3 levels of invariance: configural, metric, and scalar. The model fit between nested models was compared using χ^2^ difference testing.

**Results:**

Invariance tests indicated that the HFIAS had configural, metric, and scalar invariance across different groups of Ghanaian and South African youth. Model fit statistics across all invariance levels indicated good fit of our hypothesized model with the observed data. χ^2^ difference testing results were not statistically significant across all nested models.

**Conclusions:**

Food insecurity, as measured by the HFIAS, meant the same thing for different groups of Ghanaian and South African youth. Evidence of invariance means that the HFIAS scores could be interpreted as identical across youth groups in our study.

## Introduction

Food insecurity, defined as lack of access by individuals to adequate resources necessary to obtain appropriate foods for a nutritious diet,^1^ is primarily measured using scales or sets of questionnaire items that ask respondents a series of questions about their experiences with obtaining food regularly. The use of multiple-item, experience-based food insecurity scales is commonly considered a best practice due to the multidimensionality and complexity of food insecurity. Additionally, the use of multi-item scales is often viewed as psychometrically reliable and valid, compared to single-item measures that may not adequately assess latent constructs or variables that cannot be observed directly, such as food access.^2^ Thus, the use of multi-item experience-based food insecurity scales such as the Household Food Insecurity Access Scale (HFIAS) and the Food Insecurity Experience Scale (FIES) is common in assessing food insecurity in research and practice. Information gathered from these multi-item scales has been used to assess food insecurity prevalence,^3,4^ changes in food insecurity over time,^5^ and risk factors and consequences of food insecurity.^4,6^ These scales have also been used to measure food insecurity to identify food-insecure populations and guide the implementation and monitoring of food assistance programs.^7,8^

Additionally, experience-based food insecurity scales have been used to compare the prevalence of food insecurity and differences in risk factors across groups such as gender, age, and education level.^9,10^ For example, a 2017 study found that 41% of children (or an estimated 605 million children) younger than 15 years lived in moderately or severely food-insecure households across 147 countries and territories.^10^ Further, risk factors associated with food insecurity differ across youth groups, including gender and age.^11-13^ For example, social norms assign women with subordinate roles to men, resulting in gender-biased household food allocation, with girls and women receiving smaller portions or a less diverse diet.^12,14^ Youth’s age also affects the risk of food insecurity, with older youth more likely to experience food insecurity than younger youth.^9^

Cross-national comparisons of food insecurity prevalence and its effects have also utilized multi-item measures.^10,15^ When scores collected from distinct groups are interpreted in the same way, researchers and practitioners assume that identical scores represent the same level of food insecurity for members of different groups or populations. However, scores should not be assumed or interpreted as identical across groups because the nature and magnitude of relationships between items that comprise the food insecurity scale and the latent phenomenon of food insecurity may differ.^16,17^ Lack of evidence suggesting that the same underlying construct of food insecurity is being measured across groups or populations may lead to inaccurate results.^18^ In turn, these erroneous results could misidentify at-risk and food-insecure groups as having adequate food access, obscure correlations between food insecurity and health outcomes among different subgroups, or be adopted as guidelines for food assistance programs that are irrelevant to target populations. Thus, establishing whether food insecurity scale scores can be interpreted as identical across groups has implications for both research and practice.

Tests of cross-group similarities in the relationships between latent constructs and scale items are tests of measurement invariance. The magnitude of measurement invariance is further underscored by evidence showing significant cross-group differences in HFIAS scores between men and women from the same household. Prior research found that adult men and women living in the same household responded differently to food insecurity questions due to their divergent food-related roles and responsibilities.^19^ As only one respondent is typically required to describe a household’s experiences of food insecurity, demonstrating that different respondents—either the adult male, adult female, or youth—understand food insecurity questions and interpret response options similarly is critical. Evidence of invariance should be recognized before any further analyses, such as tests of hypothesized associations and impact evaluation of food assistance programs, are conducted. The HFIAS has not been validated with youth samples, although its psychometric properties have been previously tested and established when used with adults from low- and middle-income countries (LMICs).^20-22^ As more research studies on youth food insecurity become available, it is crucial to demonstrate the validity of the HFIAS (and other experience-based food insecurity scales) with youth, who are different from the original adult populations surveyed during scale development and validation.

We developed this study to address 2 prominent measurement issues. First, we evaluated the validity of the HFIAS when used with adolescents and young adults in 2 LMICs with a high prevalence of food insecurity—Ghana and South Africa. Prior studies have not validated the HFIAS using data collected from youth respondents, though the scale has been previously used to examine the association of food insecurity and health outcomes among adolescents and young adults.^6,23,24^ Research has also shown that adolescents comprehend food insecurity and accurately report their food insecurity experiences.^25,26^ Second, we assessed measurement invariance of the HFIAS using the same sample of adolescents and young adults from Ghana and South Africa. Although several studies have used confirmatory factor analysis (CFA) to demonstrate the construct validity of HFIAS,^27,28^ we are not aware of any published study that tested measurement invariance of HFIAS using CFA, a type of structural equation modeling, in either youth or adults. More importantly, given the implication of measurement invariance for research and practice, we examined whether the HFIAS scores could be interpreted as identical across various youth groups. Our research objective was to evaluate whether the same underlying construct of food insecurity, as measured by the HIFAS, was being assessed across various youth groups.

## Methods

### Study Design

We analyzed cross-sectional quantitative data from 2 youth-focused economic-strengthening projects conducted in Ghana and South Africa. The institutional review boards at the University of Ghana, the University of Johannesburg, and the University of North Carolina at Chapel Hill approved the original study protocols. In both projects, research staff met with prospective participants (and their caregivers, if a participant was a minor) to explain the study. If interested in participating, the research staff scheduled a follow-up meeting to obtain consent. For non–English-speaking persons, the information sheet and consent form were translated into local languages. Recruitment was conducted at schools (Ghana) and employment training sites (South Africa). Informed consent (and assent for those younger than 18 years at the time of data collection) was obtained from all individual participants in the study. For minor participants in the Ghana project, we first obtained consent from an adult caregiver. After receiving adult informed consent, we collected the assent of the participant. Participants in the South Africa project were aged 18 years or older at the time of data collection.

### Sample and Study Setting

Our study sample comprised 1437 and 4165 young South Africans and Ghanaians, respectively. We limited our study sample to youth between the ages of 15 and 24, consistent with the United Nation’s youth definition.^29^

#### Ghana

The Ghanaian sample was a subset of youth who participated in a financial inclusion project for school-going youth. The details of the original project are described in the endline report.^30^ The original project was implemented in 8 of 10 administrative regions in Ghana. A multistage sampling method was used to identify the schools and students within the selected schools that comprised the study. After identifying the 8 administrative regions in Ghana where the financial institution (FI) partner operated in 2011, 100 public junior high schools (JHS) were randomly selected from an eligible pool of 581 JHS within the FI’s service area. While the 100 schools were spread across 8 of 10 administrative regions in 2011, 63 of 100 junior high schools were in 3 regions, namely, Eastern (24 schools), Greater Accra (21 schools), and Ashanti (18 schools). At each selected JHS, the school enrollment list was used to select eligible youth participants based on their age (12-14 years old at the time of baseline data collection) and enrollment status (currently in school). Endline data were collected in 2014. The endline sample comprised 4165 youth between the ages of 15 and 24 years.

#### South Africa

The South African sample was a subset of youth who participated in a youth employability and financial capability project implemented in all 9 South African provinces. The details of the original project are described in its baseline report.^31^ Data were from the baseline survey of the project, collected in 2015. The project used a cluster-randomized design with a final cluster sample of 44 training sites. The 44 sites represented all training sites that the 8 youth-focused implementing organizations administered. The enrollment list at each training site was used to assess eligibility and to select youth participants. Inclusion criteria included age (≥ 18 years old), not currently employed, not currently in school, not currently enrolled in a training program, and a citizen of South Africa. The average number of youth recruited and enrolled per site was 43. While the baseline sample included 1993 participants, 1437 youth were between 15 and 24 years old.

### Data Collection and Sources

Data were collected using interviewer-administered questionnaires. The survey questionnaires in both countries included information on demographic, socioeconomic, educational, and financial characteristics of youth, their parents, and their households. All data, including household food insecurity, analyzed in this study were reported by youth. In Ghana, household food insecurity was only measured at endline (2014). In South Africa, we used the household food insecurity data collected at baseline (2015).

### Measures and Variables

#### Food insecurity

We evaluated the construct validity and measurement invariance of an adapted version of the HFIAS. The HFIAS, an experience-based food insecurity scale, has been used to study food insecurity in LMICs, including assessing the prevalence of food insecurity.^4,9,21,22,24,27,32^ Previous qualitative research informed the development of HFIAS by providing insight into how households experienced food insecurity.^33^ The original HFIAS includes 9 “occurrence” questions that ask whether a specific condition associated with the experience of food insecurity ever occurred during the previous 4 weeks (or 30 days). For participants with an affirmative response to the occurrence question, a follow-up question asks them how often the reported condition occurred during the previous 4 weeks, with responses ranging from rarely (ie, once or twice in the past 4 weeks), sometimes (ie, 3-10 times in the past 4 weeks), and often (ie, more than 10 times in the past 4 weeks).^32^ While the original HFIAS includes 9 “occurrence” and 9 “frequency-of-occurrence” questions, our adapted version combined the occurrence and frequency-of-occurrence questions into 1 item, resulting in 9 questions that capture behavioral and psychological manifestations of inadequate food access and their frequency of occurrence (see Supplementary material). These adaptations did not change the wording or language of the HFIAS. The adapted items asked youth how frequently a specific condition associated with the experience of food insecurity occurred during the previous 4 weeks, with response options ranging from never (0 times in the past 4 weeks) to often (more than 10 times in the past 4 weeks). Responses to the 9 items are summed, with scores ranging from 0 to 27. Higher scores represent an increasing level of severity of food insecurity.

As a categorical variable, households are classified as food secure, mildly food insecure, moderately food insecure, or severely food insecure. Households that respond affirmatively to the more severe food-related behaviors (or experience them more frequently) are classified as severely food insecure. For example, a food-secure household does not experience inadequate access to food or just experiences worry, but rarely, that is, once or twice in the past 4 weeks. A mildly food-insecure household worries about 1 or more of the following conditions with a corresponding frequency of occurrence: not having enough food sometimes or often, and/or is unable to eat preferred foods, and/or eats a more monotonous diet than desired and/or some foods considered undesirable, but only rarely. A mildly food-insecure household does not reduce quantity (either size or number of meals) nor experience any of the 3 most severe food insecurity conditions (running out of food, going to bed hungry, or going a whole day and night without eating). Further, a moderately food-insecure household forgoes better tasting or higher quality foods more frequently. Moderately food-insecure households eat a monotonous diet or eat undesirable foods sometimes (ie, 3-10 times in the past 4 weeks) or often (ie, more than 10 times in the past 4 weeks). Although it involves cutting back on quantity by reducing the size of meals or number of meals, rarely or sometimes within the past 4 weeks before data collection, moderate food insecurity does not result in any of the 3 most severe conditions. On the other hand, severe food-insecure households have started cutting back on meal size or the number of meals often and/or experiences any of the 3 most severe conditions (running out of food, going to bed hungry, or going a whole day and night without eating), regardless of frequency of occurrence. In other words, any household that experiences one of these 3 conditions even once in the last 4 weeks (or 30 days) is considered severely food insecure.^32^

#### Youth group variables

We examined invariance of the HFIAS across different groups, including age group (middle adolescence [15-17 years], late adolescence [18-21 years], or emerging adulthood [22-24 years]), sex (male or female), and receipt of child support grant [CSG] (yes, no, or do not know). Age and sex were groups in Ghana and South Africa, whereas CSG was only in South Africa.

### Analysis

Measurement invariance testing involves comparing the fit of a succession of nested models, each with more equality constraints on parameters across groups than the previous model.^34^ Measurement invariance focuses on the measurement parameters instead of predictive relationships among latent variables. Although there are various approaches for testing measurement invariance, we conducted measurement invariance testing using multiple-group CFA.^35,36^ Our selection of CFA over other approaches was based on suggestions in the literature^37,38^ and prior validation studies of the HFIAS.^27,28^

We followed the steps outlined by Bowen and Masa (2015) to assess measurement invariance of the HFIAS across 5 groups of youth (gender and age group in Ghana, gender, age group, and receipt of CSG in South Africa).^39^ Given that HFIAS items are measured at the ordinal level, the parameters of interest in our invariance testing are factor loadings, thresholds, and residual variances.^34^ However, the levels of invariance testing with ordinal data are the same as general invariance testing procedures, which comprise configural, metric, scalar, and strict levels of invariance. We did not assess the fourth level of invariance, strict invariance, in which residual variances are constrained to be equal across groups (in addition to factor structure, loadings, and thresholds) because this level of invariance is rarely achieved in practice.^39^ In turn, strict invariance is considered an unreasonable expectation across groups. We conducted sequentially the 4 steps described in the next paragraph for each of the 5 cross-group comparisons. All analyses were conducted using Mplus 8.1.

First, we examined the construct validity of the HFIAS with the overall youth sample by country. We hypothesized that all 9 HFIAS items would load onto a single factor as suggested by the scale developers and consistent with previous research.^32,40,41^ We tested alternative models, including a 2-factor structure. However, the 1-factor model had the best fit compared to the alternative models. We used the resulting unidimensional model as our baseline model for invariance testing. We confirmed the same unidimensional model for each group by country, for a total of 11 CFAs. Second, we used the group-specific baseline model and assessed for *configural* (also known as form or pattern) invariance. Configural invariance requires a factor model with the same pattern of factor loadings across groups.^37^ In this level, we tested whether the same HFIAS items loaded onto the same factors across groups using multiple-group CFA. The configural test’s objective was to determine whether the same (HFIAS) items measure household food insecurity across multiple groups of youth. We assessed whether the unconstrained multiple group model met fit criteria. If the fit criteria were met, we proceeded to the next level of invariance testing. If fit criteria were not met, invariance testing did not continue because the hypothesized factor model (in this case, 1-factor HFIAS) was not acceptable for 1 or both groups.

Third, we assessed *metric* or *weak* invariance. Scales with metric invariance have statistically equivalent factor loadings across groups and have the same pattern of factor loadings across groups.^37^ The objective was to determine whether the latent construct, food insecurity, was defined consistently across groups, that is, factor loadings of the HFIAS items were equivalent across groups.^42^ Metric invariance suggests that the construct of interest has the same meaning to youth participants across groups. Alternatively, noninvariant factor loadings signify that the HFIAS indicators have different relationships to the latent construct across groups and that the construct has different meanings to youth participants across groups.

Fourth, we assessed *scalar* or *strong* invariance. This third level of invariance is defined by the presence of invariant thresholds for ordinal HFIAS data, in addition to invariant loadings and factor structure. In a threshold model, ranges of normalized scores from an underlying continuous-level latent variable correspond to ordinal response options.^16^ The ranges are defined by thresholds. In the adapted HFIAS version, 4 response values (0, 1, 2, 3) mean 3 thresholds. The objective was to determine whether the thresholds were noninvariant or equivalent across different groups of youth. Scalar invariance implies that differences in scale scores are caused by differences in true levels of the underlying construct, not other causes.^16,37^ Scales with scalar invariance are considered adequately invariant for most practice and research purposes. If scalar invariance is demonstrated, researchers can compare factor means, variances, and covariances across groups and can evaluate hypothetical directional relationships.^37^

#### Estimation and model fit criteria

We used robust weighted least squares (WLS) as the estimator due to the ordinal-level data comprising the HFIAS.^43,44^ In Mplus, we used the recommended robust WLS estimator, means and variance-adjusted WLS.^44^ While χ^2^ is a recommended measure of fit, our relatively large sample size (ie, models with 400 or more cases) means that the χ^2^ is often statistically significant.^45^ For this reason, we used additional fit indices, such as root mean square error of approximation (RMSEA), comparative fit index (CFI), Tucker-Lewis index (TLI), and standardized root mean square residual (SRMR) for assessing model fit at each level of invariance.^45^ As indicators of good fit, we used a CFI and TLI of ≥ 0.95, the RMSEA point estimate of ≤0 .06 and upper CI of ≤ 0.06, and a value ≤ 0.08 for SRMR.^46,47^ However, due to a lack of consensus about goodness-of-fit indices and recommended cutoff values for assessing fit,^48,49^ we used these cutoff values considering the limitations noted in the literature.^50,51^ Consistent with published studies,^48,52^ we examined multiple indices to determine model fit.

#### χ^2^ difference tests and reduction in model fit

Invariance is supported when the decrease in fit is not statistically significant.^17^ We evaluated whether model fit decreased significantly by using the χ^2^ difference tests, which obtain the difference of the χ^2^values of 2 nested models (ie, configural and metric models and metric and scalar models) and the difference of the degrees of freedom. If the χ^2^_diff_ value is insignificant (*P* > .05), both models fit equally well statistically, and the model with additional equality constraints (metric or scalar) can be accepted.^53^ Some researchers suggest comparing only the decrement in fit between the configural and scalar model as factor loadings and thresholds should only be freed or constrained in tandem.^54^ However, we included the metric model in our illustration because of the advantages of testing factor loadings and thresholds separately, such as identifying noninvariant thresholds and loadings separately.^39^

## Results

### Sample Characteristics

Thirty-one percent of the Ghanaian sample was from food-secure households (n = 1293). The rest of the Ghanaian sample reported experiencing food insecurity (n = 2872). Among Ghanaian youth from food-insecure households, most (51%) experienced severe food insecurity, and 36% and 13% reported moderate and mild food insecurity, respectively. Mean HFIAS score was 5.44 points (SD = 5.83). Females accounted for 51% of the sample. Mean age was 16.48 years (SD = 1.92). Sixty-nine percent of Ghanaian youth were in their late adolescence (18-21 years old), while 15% were in their middle adolescence (15-17 years old). The remaining 16% were young adults (22-24 years old).

A similar pattern of high prevalence of food insecurity was observed in the South African data, with 83% of youth (n *=* 1180) reported inadequate food access. Among youth from food-insecure households, 58% experienced severe food insecurity, 23% moderate food insecurity, and 19% mild food insecurity. Mean HFIAS score was 6.80 (SD = 6.47). South African youth were older (mean age = 21.91, SD = 1.77) compared to Ghanaian youth. The study sample comprised more females (61%) and young adults (67%) than males and adolescents, respectively. Twenty-eight percent reported receiving the CSG during their childhood.

### Confirmatory Factor Analysis

Results indicated adequate fit of a 1-factor food insecurity model with the observed data from Ghanaian youth (χ^2^[27, N *=* 4165] = 1118.83, *P* < .001, RMSEA = 0.099 [90% CI, 0.094-1.04], CFI = 0.972, TLI = 0.963, SRMR = 0.048). Similarly, the South African results based on a 1-factor food insecurity model were similar (χ^2^[27, N *=* 1434] = 655.43, *P* < .001, RMSEA = 0.128 [90% CI, 0.120-1.37], CFI = 0.978, TLI = 0.971, SRMR = 0.041). In both countries, all factor loadings were statistically significant (*P* < .001). The percentages of variance or (values) in each observed item explained by the measurement model ranged from 0.52 to 0.68 in Ghana and 0.52 to 0.83 in South Africa. Although RMSEA values did not meet our predetermined fit criteria, cutoffs for other fit indices were met.

### Measurement Invariance

#### Baseline model for each group

Table 1 lists the model fit statistics for the baseline model for each youth group. As an initial step, we identified the best-fitting model for each group. Based on prior empirical work on HFIAS and our analysis of alternative models,^40,41^ we tested a 1-factor food insecurity model. Model fit indicated an adequate fit of the 1-factor model for each group. The CFI, TLI, and SRMR values met the cutoff criteria, whereas RMSEA values were slightly over the cutoff. Published studies have noted that tests of the configural model can be conducted even with marginally adequate baseline models for each group.^55^

**Table 1.** Model Fit Statistics for Baseline Models Using Confirmatory Factor Analysis.^a^

Youth groups	χ^2^	RMSEA (90% CI)	CFI	TLI	SRMR
South Africa					
Gender					
Male	277.86^b^	0.129 (0.116-0.143)	0.979	0.972	0.043
Female	359.15^b^	0.120 (0.109-0.131)	0.981	0.975	0.040
Age group					
Late adolescence	516.07^b^	0.137 (0.127-0.148)	0.977	0.970	0.045
Emerging adulthood	151.54^b^	0.099 (0.084-0.115)	0.984	0.978	0.035
Child support grant					
No	347.25^b^	0.123 (0.112-0.135)	0.983	0.978	0.038
Yes	205.09^b^	0.129 (0.113-0.146)	0.973	0.964	0.049
Do not know	123.17^b^	0.127 (0.105-0.150)	0.978	0.970	0.053
Ghana					
Gender					
Male	584.05^b^	0.100 (0.093-0.108)	0.972	0.963	0.046
Female	547.94^b^	0.095 (0.089-0.103)	0.973	0.964	0.050
Age group					
Middle adolescence	144.38^b^	0.082 (0.070-0.096)	0.979	0.972	0.047
Late adolescence	778.85^b^	0.098 (0.092-0.104)	0.971	0.961	0.050
Emerging adulthood	256.71^b^	0.115 (0.102-0.128)	0.969	0.958	0.052

Abbreviations: CFI, comparative fit index; RMSEA, root mean square error of approximation with 90% CI; SRMR, standardized root mean square residual; TLI, Tucker-Lewis index.

^a^ χ^2^ difference test (level of significance .05).

^b^ *P* < .001.

#### Configural models

Table 2 presents the fit statistics for each cross-group comparison at each level of invariance testing. We assessed the overall model fit to assess whether configural invariance holds. Three of 5 indices (CFI, TLI, and SRMR) met our prespecified model fit criteria. Results indicated that all 9 items comprising the HFIAS measured the same unidimensional food insecurity construct across administrations or multiple youth groups in Ghana and South Africa.

**Table 2.** Fit Statistics for Each Cross-Group Comparison at Each Level of Invariance Testing.^a^

Models compared	χ^2^ (*P*)	RMSEA (90% CI)	CFI	TLI	SRMR
Configural					
Gender (Ghana)	1130.52^b^	0.098 (0.093-0.103)	0.973	0.964	0.048
Gender (South Africa)	638.84^b^	0.124 (0.115-0.132)	0.980	0.974	0.041
Age group (Ghana)	1159.87^b^	0.098 (0.093-0.103)	0.972	0.963	0.050
Age group (South Africa)	675.99^b^	0.127 (0.118-0.136)	0.979	0.971	0.042
Age group (South Africa)	655.29^b^	0.123 (0.115-0.132)	0.981	0.975	0.043
Metric					
Gender (Ghana)	1109.25^b^	0.090 (0.085-0.095)	0.973	0.969	0.048
Gender (South Africa)	658.71^b^	0.116 (0.108-0.124)	0.980	0.977	0.041
Age group (Ghana)	1123.34^b^	0.087 (0.083-0.092)	0.973	0.970	0.050
Age group (South Africa)	674.22^b^	0.118 (0.110-0.126)	0.979	0.976	0.042
Child support grant (South Africa)	672.00^b^	0.113 (0.105-0.121)	0.981	0.979	0.044
Scalar					
Gender (Ghana)	925.56^b^	0.072 (0.068-0.076)	0.978	0.980	0.048
Gender (South Africa)	587.29^b^	0.095 (0.088-0.103)	0.983	0.984	0.042
Age group (Ghana)	918.42^b^	0.066 (0.062-0.070)	0.980	0.983	0.050
Age group (South Africa)	603.71^b^	0.096 (0.089-0.104)	0.982	0.984	0.042
Child support grant (South Africa)	589.41^b^	0.087 (0.080-0.094)	0.985	0.988	0.044

Abbreviations: CFI, comparative fit index; RMSEA, root mean square error of approximation with 90% CI; SRMR, standardized root mean square residual; TLI, Tucker-Lewis index.

^a^ χ^2^ difference test (level of significance .05).

^b^ *P* < .001.

#### Metric models

Table 3 lists the results of the χ^2^ difference tests. For each cross-group comparison, we compared the fit of the model with all factor loadings constrained across groups to the fit of the configural model. The change in χ^2^ per change in degrees of freedom (df) was nonsignificant for each cross-group comparison, indicating that the groups’ factor loadings were statistically equivalent. For example, when we compared the fit of the model across the 2 age groups in South Africa (late adolescence and emerging adulthood), the change in χ^2^ per change in df was nonsignificant, Δχ^2^(8) = 5.544, *P* = .70. Similarly, the model fit did not significantly decrease when we compared the fit of the metric and configural models across the 3 age groups (middle adolescence, late adolescence, and emerging adulthood) in Ghana, Δχ^2^(16) = 18.957, *P* = .27. Furthermore, we assessed the overall model fit to test whether metric invariance holds (see Table 2). Three of 5 indices (CFI, TLI, and SRMR) met our prespecified model fit criteria. While they did not meet our prespecified criteria, RMSEA values slightly improved from configural to metric models. We retained the constrained factor loadings with evidence of metric invariance and proceeded to a scalar invariance test.

**Table 3.** Results of χ^2^ Difference Tests Comparing Nested Models.^a^

Models compared	χ^2^	df	*P* value
Gender (Ghana)			
Metric against configural	11.20	8	.19
Scalar against configural	34.36	25	.10
Scalar against metric	23.97	17	.12
Gender (South Africa)			
Metric against configural	15.02	8	.06
Scalar against configural	38.48	25	.04
Scalar against metric	24.97	17	.10
Age group (Ghana)			
Metric against configural	18.96	16	.27
Scalar against configural	50.38	50	.46
Scalar against metric	32.33	34	.55
Age group (South Africa)			
Metric against configural	5.54	8	.70
Scalar against configural	25.87	25	.41
Scalar against metric	19.43	17	.30
Child support grant (South Africa)			
Metric against configural	18.90	16	.27
Scalar against configural	41.84	50	.79
Scalar against metric	24.65	34	.88

Abbreviation: df, degrees of freedom.

^a^ χ^2^ difference test, *P* value (level of significance .05).

#### Scalar models

The fit of a model with factor loadings and item thresholds constrained to be equal across groups was compared to the fit of the metric model for each cross-group comparison (age and gender in Ghana and South Africa, and receipt of CSG in South Africa). The change in χ^2^ per change in df was nonsignificant across each cross-group comparison, indicating that the groups’ thresholds were statistically equivalent. Using age group as an example, we compared the fit of the model across the 2 age groups in South Africa (late adolescence and emerging adulthood), the change in χ^2^ per change in df was nonsignificant, Δχ^2^(17) = 19.433, *P* = .30. Similarly, model fit did not significantly decrease when we compared the fit of the scalar and metric models across the 3 age groups (middle adolescence, late adolescence, and emerging adulthood) in Ghana, Δχ^2^(34) = 32.328, *P* = .55. Furthermore, we assessed the overall model fit to examine whether scalar invariance holds. Fit statistics for each cross-group comparison at the scalar level of invariance testing are presented in Table 2. Three of 5 indices (CFI, TLI, and SRMR) met our prespecified model fit criteria. Also, RMSEA values improved from less constrained (metric) to more constrained (scalar) models. The RMSEA values met our prespecified fit criteria for the Ghanaian model (RMSEA = 0.066, 90% CI, 0.062-0.070). We retained the constrained factor loadings and item thresholds due to a nonsignificant difference in model fit, suggesting that HFIAS had scalar invariance across all cross-group comparisons evaluated in this study.

## Discussion

Overall, invariance tests indicated that the HFIAS had configural, metric, and scalar invariance across various Ghanaian and South African youth groups. In other words, food insecurity, as measured by the HFIAS, meant the same thing for different groups of Ghanaian and South African youth. First, the configural invariance finding suggests that the 1-factor model (or unidimensional structure) of the HFIAS applies to young men and young women in Ghana and South Africa, adolescents and young adults in Ghana and South Africa, and youth who received, did not receive, or did not know whether they received a CSG in South Africa. Second, the metric invariance suggests that individual HFIAS items have similar weights and are equally salient to the construct of food access for all youth groups in our study. Third, the scalar invariance indicates that the similar true levels of the HFIAS correspond to differences in true levels of the underlying construct (food access) across various youth subgroups in our study. Scalar invariance, with factor loadings and thresholds constrained to be equal across groups, is required to interpret scores equivalently across groups.^37,56^ Thus, our study’s mean HFIAS scores can be validly compared and meaningfully interpreted as the same construct across young men and young women in Ghana and South Africa, adolescents and young adults in Ghana and South Africa, and youth who received, did not receive, or did not know whether they received a CSG in South Africa. We can validly compare HFIAS scores because our finding of measurement invariance provides evidence that our measure of food access is interpreted in a conceptually similar manner by youth participants representing different genders, age groups, and socioeconomic status (as measured by receipt of CSG). This interpretation means that variations in the prevalence of food insecurity among adolescent Ghanaian boys and girls and young South African adult men and women in our study reflect true differences in levels of food access across genders and age groups.

Conversely, if we did not find HFIAS to be invariant between, for example, younger and older Ghanaian youth, meaningful comparisons of scores between age groups are questionable. The use of noninvariant HFIAS raises 2 issues that weaken the validity of findings comparing food insecurity between younger and older Ghanaian youth. First, the noninvariance of loadings and thresholds results in incorrect regression parameters, which may bias our findings and interpretation of observed differences about the relationship of food insecurity and youth outcomes based on age.^18^ Second, noninvariant HFIAS might be measuring different constructs in younger Ghanaian adolescents, older adolescents, and young adults in our study, although the same HFIAS instrument is used to assess food insecurity.^48^ For example, Ghanaian youth may differ in their living arrangements, with the younger youth living with their parents or caregivers, whereas the older youth living independently or with nonfamily members. Potential differences in living arrangements might result in scores that are not comparable across the 2 groups, even if the construct has the same meaning in both cultures.^33^

Invariance of the HFIAS does not suggest that there are no differences in food access between the various groups of youth in our study. However, invariance heightens the validity of cross-group comparisons because the measure used to assess food insecurity across groups is empirically established to be understood and interpreted in the same way by different groups of youth. Additionally, the latent variable representing HFIAS scores can be used in hypothesis testing across various youth groups. For example, studies that used the same Ghana and South Africa data to examine the moderating effect of gender on the relationship of food insecurity and health outcomes validly compared mean HFIAS scores among young men and young women.^9,24^

Construct validation is a valuable yet often ignored step when using scales initially developed in different settings or with a different population. Even fewer are tests of measurement invariance to justify cross-group comparison of mean scores. In the hierarchy of measurement models, a scale’s construct validity must be demonstrated before proceeding to a test of cross-group similarities or measurement invariance.^57^ When researchers are interested in examining group differences by comparing mean scale scores derived from HFIAS or other multi-item food security measures, invariance must be provided for each cross-group comparison. If there is no evidence of invariance, similar true levels of food insecurity may correspond to other causes unrelated to differences in true levels of the underlying construct. For example, cross-group differences might be due to different response choices across groups on the latent (food insecurity) variable indicators. Thus, it is inaccurate to assume that, for example, young women respond to food insecurity questions in the same way as their male counterparts. Prior research has shown that males and females from the same household respond differently to household food insecurity questions.^19^ While such differences may be due to the unreliability of individual items, it underscores the importance of assessing whether responses are comparable across groups and that results are due to differences in true levels of the underlying construct.

Findings should be interpreted in the context of study limitations. First, we examined HFIAS invariance across a limited number of groups. The HFIAS may be noninvariant across other groups that were not explored in this study. For example, our final scalar model might not hold across time (eg, pretest and posttest) or across youth grouped by residence (eg, rural and urban). We did not examine cross-national or cross-cultural invariance by comparing Ghanaian and South African youth. Instead, we sought to explore group differences within countries in order to control for extraneous influences.^58^ Second, our findings may not be generalizable to all youth in Ghana and South Africa due to the original projects’ sampling design. Third, although the HFIAS can capture different food insecurity conditions (eg, from mild to severe food insecurity) and psychosocial manifestations of anxiety, stress, and uncertainty related to food access, respondents may not be the appropriate household member to depict the household’s experience of food insecurity accurately. The HFIAS might under- or over-report food insecurity in the households. Similarly, youths’ perception of their own food insecurity experience may not represent other household members,^19^ though youth can reliably report food insecurity within their household.^25^

### Implications and Future Research

Our results indicate that the HFIAS measures the same underlying food insecurity construct in the same way across various youth groups in this study. This multi-group invariance indicates that comparing scores across youth groups by sex, age group, and CSG receipt is warranted. The HFIAS was initially developed for the primary food preparer in a household and has not been validated with youth populations. This study is one of the first to assess the construct validity and evaluate the ability of HFIAS to assess true differences in HFIAS scores between youth groups. The HFIAS is an experience-based scale like other food insecurity scales such as the FIES. Although FIES and HFIAS capture various behavioral and psychological manifestations of inadequate food access, one advantage of HFIAS is its 4-point response options that may allow youth to better discriminate the frequency of their experiences of food insecurity conditions, compared to the FIES’ binary response options (yes or no). Research is needed to establish whether food insecurity experienced by adolescents and young adults is validly assessed by the HFIAS, FIES, and other experience-based scales. However, HFIAS, FIES, and other experience-based scales do not quantify food consumption, nor do they assess diet quality. Thus, research is needed with youth participants to compare experience-based food insecurity scales and food consumption and diet quality measures to establish validity and comparability before testing structural relationships. Future studies should also assess measurement invariance among other groups of youth, including cross-cultural and longitudinal comparisons. These inquiries will increase evidence of the HFIAS’s validity and invariance. Researchers and practitioners must consider the measurement invariance of experience-based food insecurity scales to accurately estimate prevalence and relationships between constructs.

## Supplemental Material

Supplemental Material, sj-docx-1-fnb-10.1177_03795721211019634 - Invariance of the Household Food Insecurity Access Scale Across Different Groups of Adolescents and Young Adults Click here for additional data file. Supplemental Material, sj-docx-1-fnb-10.1177_03795721211019634 for Invariance of the Household Food Insecurity Access Scale Across Different Groups of Adolescents and Young Adults by Rainier Masa and Anjalee Sharma in Food and Nutrition Bulletin
