# Supplementary material for: Invariance of the Household Food Insecurity Access Scale Across Different Groups of Adolescents and Young Adults
Source: Food Nutr Bull. 2021 Jun 15;42(3):437–50. doi: 10.1177/03795721211019634 (PMC8442126; doi:10.1177/03795721211019634)
Supplement: Supplemental Material, sj-docx-1-fnb-10.1177_03795721211019634 - Invariance of the Household Food Insecurity Access Scale Across Different Groups of Adolescents and Young Adults [file sj-docx-1-fnb-10.1177_03795721211019634.docx]

*Appendix* A: Adapted HFIAS

|  | Item | Response Options |
| --- | --- | --- |
| 1. | In the past four weeks, did you worry that your household would not have enough food? | 0=Never (zero times in the past four weeks)  1=Rarely (once or twice in the past four weeks  2= Sometimes (three to ten times in the past four weeks)  3=Often (more than ten times in the past four weeks) |
| 2. | In the past four weeks, were you or any other household member not able to eat the kinds of food you preferred because of a lack of resources? | 0=Never (zero times in the past four weeks)  1=Rarely (once or twice in the past four weeks  2= Sometimes (three to ten times in the past four weeks)  3=Often (more than ten times in the past four weeks) |
| 3. | In the past four weeks, did you or any household member have to eat a limited variety of foods due to lack of resources? | 0=Never (zero times in the past four weeks)  1=Rarely (once or twice in the past four weeks  2= Sometimes (three to ten times in the past four weeks)  3=Often (more than ten times in the past four weeks) |
| 4. | In the past four weeks, did you or any household member have to eat some foods that you really did not want to eat because of a lack of resources to obtain other types of foods? | 0=Never (zero times in the past four weeks)  1=Rarely (once or twice in the past four weeks  2= Sometimes (three to ten times in the past four weeks)  3=Often (more than ten times in the past four weeks) |
| 5. | In the past four weeks, did you or any other household member have to eat a smaller meal than you felt you needed because there was not enough food? | 0=Never (zero times in the past four weeks)  1=Rarely (once or twice in the past four weeks  2= Sometimes (three to ten times in the past four weeks)  3=Often (more than ten times in the past four weeks) |
| 6. | In the past four weeks, did you or any other household member have to eat fewer meals in a day because there was not enough food? | 0=Never (zero times in the past four weeks)  1=Rarely (once or twice in the past four weeks  2= Sometimes (three to ten times in the past four weeks)  3=Often (more than ten times in the past four weeks) |
| 7. | In the past four weeks, was there ever no food to eat of any kind in your household because of a lack of resources to get food? | 0=Never (zero times in the past four weeks)  1=Rarely (once or twice in the past four weeks  2= Sometimes (three to ten times in the past four weeks)  3=Often (more than ten times in the past four weeks) |
| 8. | In the past four weeks, did you or any household member go to sleep at night hungry because there was not enough food? | 0=Never (zero times in the past four weeks)  1=Rarely (once or twice in the past four weeks  2= Sometimes (three to ten times in the past four weeks)  3=Often (more than ten times in the past four weeks) |
| 9. | In the past four weeks, did you or any household member go a whole day and night without eating anything because there was not enough food? | 0=Never (zero times in the past four weeks)  1=Rarely (once or twice in the past four weeks  2= Sometimes (three to ten times in the past four weeks)  3=Often (more than ten times in the past four weeks) |
